# The effects of 8 weeks of dynamic hamstring stretching or nordic hamstring exercises on balance, range of motion, agility, and muscle performance among male soccer players with hamstring shortness: a randomized controlled trial

**DOI:** 10.1186/s13102-025-01216-0

**Published:** 2025-07-09

**Authors:** Elham Hosseini, Mohammad Alimoradi, Mojtaba Iranmanesh, Farzaneh Saki, Andreas Konrad

**Affiliations:** 1 Department of Sports Injuries and Corrective Exercises, Faculty of Sports Sciences, Shahid Bahonar University of Kerman, Kerman, Iran; 2 Department of Exercise Rehabilitation, Faculty of Sport Sciences, Bu-Ali Sina University, Hamedan, Iran; 3 Institute of Human Movement Science, Sport and Health, Graz University, Graz, Austria

**Keywords:** Eccentric training, Flexibility, Injury prevention, Agility, Neuromuscular adaptation, Athletic performance

## Abstract

**Background:**

This study investigated the effects of an eight weeks exercise regimen with either Nordic hamstring exercises or dynamic hamstring stretching (DHS) on balance, range of motion, agility, and muscle performance in male soccer players with hamstring shortness.

**Methods:**

Sixty participants were divided into three groups: NHE (*n* = 20), DHS (*n* = 20), and control (CO; *n* = 20). The NHE group performed Nordic hamstring exercises, the DHS group engaged in dynamic stretching, and the CO group did not receive any intervention. Assessments were conducted before and after the interventions using active knee extension test (AKET), sit-and-reach test (SR), Y-balance test (YBT), Illinois agility run test (IAT), and countermovement jump test (CMJ).

**Results:**

The results showed significant improvements in measured parameters for both the NHE (AKET: 16.16%; SR: 11.33%; YBT: 16.25%; IAT: 12.84%; CMJ: 7.28%) and DHS (AKET: 2.17%; SR: 5.51%; IAT: 4.54%; CMJ: 1.26%) groups, while no significant changes were observed in the CO group (*p* ≤ 0.001). The NHE group demonstrated superior effects compared to the DHS and CO groups, except for the countermovement jump test.

**Conclusion:**

The findings suggest that including NHE in training regimens is recommended, particularly for soccer players with hamstring shortness. Therefore, both NHS and DHS positively influenced performance, range of motion, balance, and agility. However, NHE proved to be more effective, yielding more improvements that are significant across the performance, range of motion, balance, and agility parameters.

**Trial registration:**

This trial was registered at Iranian Registry of Clinical Trials (Identifier: IRCT20230612058457N3) on April 7, 2024.

**Supplementary Information:**

The online version contains supplementary material available at 10.1186/s13102-025-01216-0.

## Background

The hamstring muscles are primarily hip extensors, with a secondary role in knee flexion due to their biarticular nature [1, 2]. The length of hamstring muscles and fascicles has a strong connection with a muscle’s capacity to contract throughout an extended range of motion and at higher acceleration. Shorter fascicles exhibit a smaller proportion of sarcomeres in series, which constrains the muscle’s excursion and contractile velocity, consequently reducing flexibility and increasing susceptibility to strain during quick or powerful movements [3–5]. Additionally, hamstring shortness, characterized by higher active or passive muscle tension, can result from muscular contraction, spasm, or postural modifications, which can lead to limited range of motion (ROM) [5, 7, 8]. Also impaired hamstring extensibility correlates with unbalanced stresses in lower limb joints, increased the probability of overuse syndromes, lower limb injuries, and low back pain [9, 10]. Because biomechanical alterations may affect stabilization of joints and efficiency in movement, providing an assessment of hamstring flexibility is essential for athletic groups. Overall hamstring muscle shortening reduces hip and knee mobility, changes running biomechanics, and increases muscular strain injuries [3, 5, 7].

Additionally, hamstring shortening can negatively impact balance, ROM, and agility, reducing athletic performance and increasing injury risk. Improved hamstring flexibility and strength enhance ROM, which optimizes running mechanics and reduces compensatory movement patterns—ultimately supporting better balance and agility [9–11]. Hamstring shortening can impair balance, ROM, and agility—critical qualities for soccer players who rely on rapid directional changes, postural stability, and explosive actions like sprinting and jumping. Improving hamstring flexibility and strength enhances these abilities by optimizing running mechanics, reducing compensatory movements, and lowering injury risk [6, 11, 12]. Concerning injuries, hamstring strain injury is the most prevalent injury in soccer, representing 12% of all injuries in high-level players [13]. This is supported by the fact that hamstring strains are among the most prevalent sports injuries, accounting for 6–29% of all injuries requiring treatment [14]. A variety of factors, such as reduced flexibility, asymmetries or deficiencies in eccentric hamstring strength, inadequate core stabilization involving the gluteal and trunk muscles, and a history of prior hamstring injuries, have been implicated in the development of hamstring injuries [15–17]. The reduction of ROM is one of the modifiable risk factors associated with hamstring injuries. Studies now support several methods for increasing ROM [3, 4].

The most popular techniques to increase ROM in the long term are strength training [18], foam rolling [19], and various types of stretching [20]. A literature review indicates that frequent stretching workouts, combined with proprioceptive neuromuscular facilitation (PNF) approaches, significantly increase hamstring flexibility in athletes [21–23]. In addition, in a review by Behm et al. (2023), they found that dynamic stretching interventions improve performance and might reduce the risk of injury [24]. However, considering the mechanism for the increase in ROM following dynamic stretching, there is a lack of evidence on musculotendinous stiffness as, for example, has been reported for static stretching [25]. On the other hand, several studies have found that Nordic hamstring exercises (NHEs) improve eccentric strength and increase ROM while also having the potential to lower the risk of injury [26, 27]. While concentric training has little or no effect on muscle eccentric strength or fascicle length, eccentric training significantly increases both eccentric strength and fascicle length, leading to greater improvements in muscular strength [28, 29]. In fact, eccentric exercise has been indicated as being an effective technique to avoid hamstring strain injuries [30], which is likely the reason why NHE is gaining popularity among athletes and coaching/medical staff [31].

Generally, hamstring shortness is prevalent in athletic populations and plays a significant role in limiting performance [6]. Thus, in order to optimize athletic performance, it is imperative that coaches and athletes properly assess and address this issue. However, there has been lack of studies on the effects of dynamic hamstring stretching (DHS) protocols and NHE on the effective variables of athlete performance. Therefore, this study was based on the hypothesis that eight weeks of either NHE or DHS protocols would significantly impact soccer players with hamstring shortness, particularly improving balance, flexibility, agility, and muscle performance. Thus, the purpose of this research was to evaluate whether 8 weeks of either NHE stretching or DHS protocols affect soccer players’ hamstring shortness related balance, flexibility, agility, and muscle performance.

## Methods

### Study design

A randomized control trial was conducted between March 2024 and May 2024. All participants attended a 2-h familiarization session to learn about the tests and fill out the consent and personal forms 48 h before the baseline assessment. After this, measurements of anthropometric characteristics were conducted. Furthermore, the dominant limb (for kicking a ball), was assigned as the test leg [32]. Prior to beginning the 8-week procedure in the current investigation, hamstring shortening in the dominant leg was confirmed in all participants using an active knee extension test (AKET) [7]. To diagnose hamstring shortening, the following cut-off values were used. For males, the active knee extension angle is more than 33.0 degrees [33]. Following confirmation of hamstring shortness in 60 participants (AKET: 36.5 ± 2.1-degree, range: 33.2 to 41.8-degree) out of 79, the participants were randomized into three groups: NHE group (*N* = 20), DHS group (*N* = 20), and control condition (CO; without stretching) (*N* = 20). In order to investigate the effect of the 8-week stretching protocols, all participants performed an AKET, sit-and-reach test, Y-balance test (YBT), Illinois agility run test, and countermovement jump (CMJ) test as pre-test and post-test evaluations. This study adheres to the CONSORT (Consolidated Standards of Reporting Trials) guidelines for the reporting of randomized controlled trials.

### Participants

Sixty male soccer players (age: 21.46 ± 2.45 years; height: 179.45 ± 6.56 cm; mass: 73.98 ± 4.38 kg) volunteered for the present research. G*Power software (Version 3.1.9.4) (repeated measure ANOVA, α = 0.05, ES = 0.41, main outcome flexibility following dynamic stretching [34]) with a statistical power of 0.9 was used to calculate the sample size of 60 soccer players. Three groups of participants were assigned: NHE group, DHS group, and control group (CO). Before the pre-test and post-test sessions, participants were asked to avoid from performing any type of intensive physical activity for at least two days. Participants were generally active in soccer; however, they were not involved in any official competitions or frequent intense physical activities during the study process. Athletes between the ages of 18 to 25 having at least three years of consistent soccer training experience (three sessions per week) were the inclusion criteria. In addition, study participants with muscular disorders or injuries to their lower extremities were excluded (according to their self-reports and medical records). Exclusion criteria also included regular drug use, a history of any cardiovascular or pulmonary diseases, or participating in massage, foam roller, hydrotherapy, or heavy physical activity protocols throughout the current procedure. Participants provided their informed consent by signing a written consent form after being advised of any potential risks associated with the current procedures. Subsequently, the included participants were divided randomly using the Random.org website to NHE, DHS, and CO groups, at a ratio of 1:1:1. A block size of 3 was used to ensure balanced group sizes throughout the study. Following the group assignment, participants underwent a baseline assessment, performed by a researcher blinded to the group assignments of the participants, in order to ensure an unbiased evaluation. The present research was accepted by the Research Ethics Committee of Bu-Ali Sina University in Hamedan, Iran (IR.BASU.REC.1402.102). The study was registered with the Iranian Registry of Clinical Trials (IRCT20230612058457N3). In addition, the principles of the Declaration of Helsinki were followed by the authors.

### Interventions

**Fig. 1:**
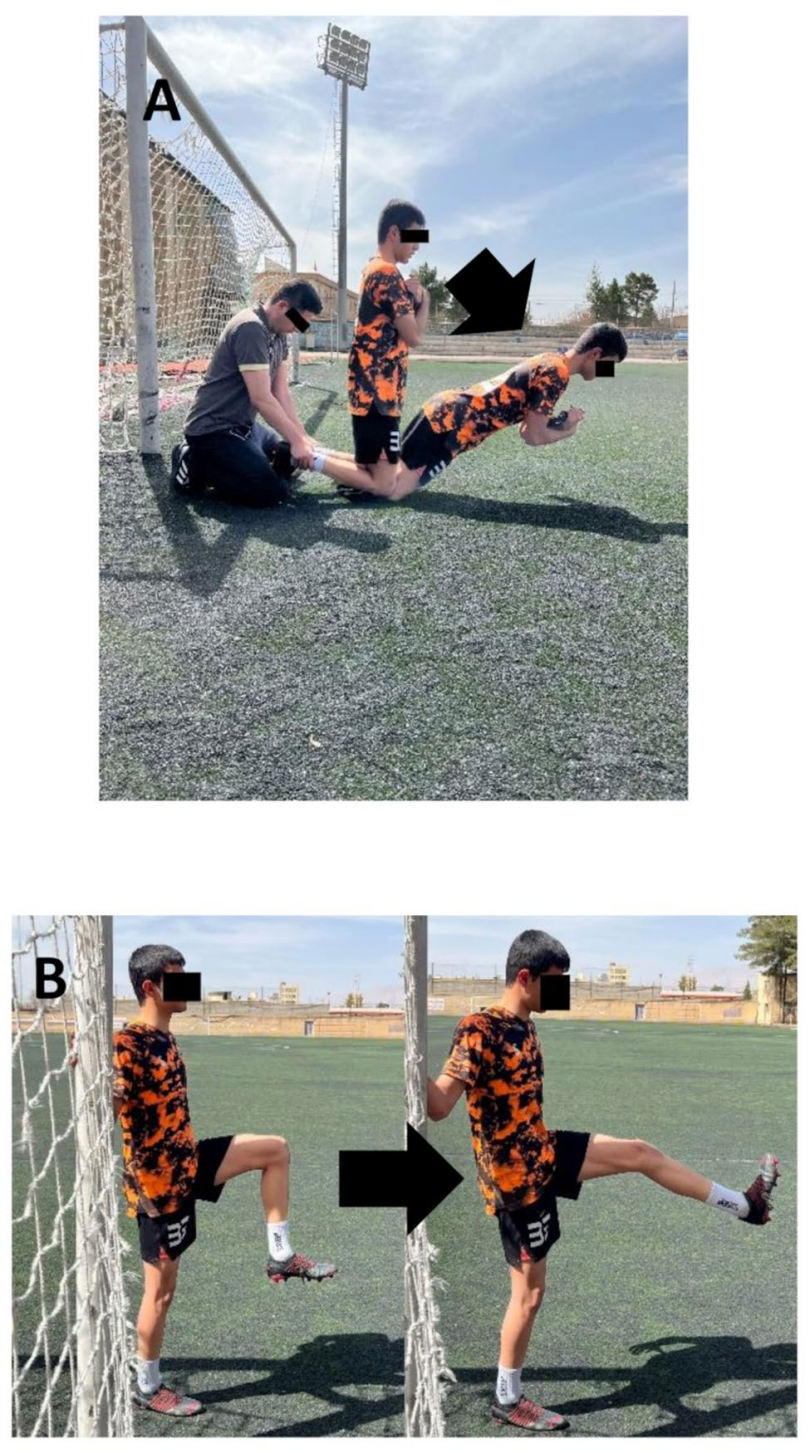
Interventions targeting the hamstring muscles: (**A**) Nordic hamstring exercise, (**B**) dynamic hamstring stretching

The exercise protocols (NHE, DHS) were performed for 8 weeks with five sessions per week. Each participant in the NHE group was positioned in a kneeled position on a padded mat and had their lower legs stabilized by a researcher (Fig. 1A). The participant was encouraged to slowly lower their torso to the floor level in a constant cadence (4 s per repetition), sustaining a rigid torso position and only moving the knee joint. The participant’s arms were flexed at the elbow joints such that the palms of the hands were facing forward at the level of the shoulder joints. The participant was allowed to use their arms in the final stages of the movement to buffer the fall. During the return to the NHE starting position, the participant used their upper limbs in order to avoid concentric actions of the hamstrings. The numbers of repetitions and sets in the training sessions (Table 1) were adjusted based on the ability of the participants, and there was a 1-min rest period between sets [26]. It should be noted that, before starting the NHE group intervention, each participant performed five trials as familiarization. Each participant in the DHS group stood erect, feet parallel, and facing front (Fig. 1B). The participant was then asked to contract their hip flexors and flex their hip joints while keeping their knees extended, resulting in their dominant leg swinging up to the anterior area of their body and stretching their hamstring muscles. Each participant completed five repetitions for each leg of the DHS exercise as familiarization, followed by performing the exercise as rapidly and strongly as possible without bouncing, in time with the rhythm of a digital metronome set to 60 beats per minute. The DHS protocol was repeated for five sets, five days per week, for 8 weeks. Each set lasted 30 s, with a 10-s rest period in between, during which the participant could rest in the original standing position [35, 36]. Finally, the participants in the CO group performed their routine exercises and did not perform a specific stretching protocol. Out of the 40 intervention sessions, the NHE group attended 39 sessions (97.5%), while the DHS group participated in all sessions (100%). Moreover, no adverse events occurred in either of the intervention groups.

**Table 1:** Details of the intervention protocols

*Nordic hamstring exercise protocol*		
Week	Sessions per week	Sets and repetitions
1	5	2 × 5
2	5	2 × 6
3	5	3 × 6–8
4	5	3 × 8–10
5–8	5	3 × 12, 10, 8 reps
*Dynamic Hamstring stretching protocol*		
1–8	5	5 × 30s (each limb)

Note: reps = repetitions

### General soccer training load

Throughout the 8-week intervention period, all participants continued their regular soccer team training routines in addition to their assigned intervention protocols. These team sessions occurred 5 times per week and lasted approximately 90 min each (Supplementary Table 1). Training sessions typically consisted of a structured warm-up, technical drills, tactical exercises, small-sided games, and general conditioning. Importantly, no additional flexibility or strength-based interventions specifically targeting the hamstrings were included in the team training. This design ensured that the observed effects could be attributed specifically to the NHE or DHS interventions, rather than confounding training variables.

### Measures

#### Y-balance test (YBT)

An OctoBalance device (Check your Motion, Albacete, Spain) was used to evaluate dynamic balance by measuring three lower limb excursion directions: anterior (YBT-A), posteromedial (YBT-PM), and posterolateral (YBT-PL). Each participant was instructed to stand on one leg in the center of the Y and extend their other leg as far as possible while maintaining balance, keeping their hands on their hips. A trial was deemed unsuccessful if the participant touched the ground while reaching, lost balance, raised both arms for stabilization, or lifted the heel of the standing leg during the movement [37]. The dominant leg was tested three times, with a 10-s passive recovery period between trials. The average performance of the three trials for the dominant leg was calculated for further analysis [38]. The mean and standard deviation for each direction were determined using the range of values from the test, normalized by the actual length of the limb. To compare athletes, the range measurement was adjusted for limb length. The normalized value was calculated by dividing the sum of the three range values by three times the limb length and then multiplying by 100 [39]. (Y-Balance, 0.681–0.908 ICC) [40, 41].

#### ROM tests

##### Sit-and-reach test

This test involves sitting on the floor with legs extended straight ahead and shoes removed. The soles of the feet are placed flat against the custom-made sit-and-reach box, with both knees locked and pressed flat to the floor. With palms facing downward and hands stacked on top of each other, the participant reaches forward along the measuring line as far as possible. It is important to ensure that both hands remain at the same level, with neither hand reaching further forward than the other [42]. (ICC = 0.98 ) [43].

##### Active knee extension test (AKET)

Hamstring shortness was assessed using the AKET. A cut-off value of more than 33 degrees was used to determine hamstring shortness [33]. Each participant was positioned supine in a comfortable position. The hip of the tested leg was flexed to 90 degrees and stabilized with a strap to ensure stability and prevent pelvic movement, with a towel placed under the spine to maintain spinal alignment. The contralateral leg remained straight and was also stabilized with a strap for measurement accuracy. The participant was then instructed to actively extend their knee until they felt significant stretching discomfort. At this point, the terminal knee extension ROM was measured using a universal goniometer. A full-circle goniometer was utilized to measure the angle of knee extension, with the fulcrum placed at the lateral condyle of the femur, the stationary arm aligned along the femur using the greater trochanter as a reference, and the movable arm aligned with the lower leg using the lateral malleolus as a reference [23]. (ICC = 0.98–0.99) [23, 44].

#### Agility and muscle performance tests

##### Illinois agility run test

The Illinois agility run test called for a field that was 10 m in length and 5 m in width. Four cones, spaced 3.3 m apart in the center of the field, indicated the start, finish, and two turning places. After hearing the word “Go”, each participant was prompted to run as fast as they could between the cones. A successful trial was one in which the participant finished the test without connecting with any cones and passed the finish line. The assignment was completed on a soccer field, and the time taken to complete the task was measured using a timing gate system (Smartspeed, Fusion Sport, Australia). During the functional assessment, all participants wore the same soccer equipment, including shoes and shirts. Three successful trials were completed with a 2-min rest interval between each trial, and the best trial was chosen for further examination ( ICC = 0.85–0.98) [45, 46].

#### Countermovement jump (CMJ) test

The CMJ test began with the participant standing straight with their hands on their hips to prevent their arms from being used during the jump. The CMJ test required a quick vertical upward movement as high as possible, followed by a rapid downward movement of the knee to about 90 degrees flexion, all in one sequence. Emphasis was given to ensure that the participant landed with a straight knee, as this is crucial for the accuracy of the jump height calculation. The My Jump 3 app for iPhone 13 was used to calculate jump height by manually selecting the take-off and landing frames from the video. The app calculates jump height using the equation (h = t^2^ × 1.22625), where h is the jump height (in meters) and t is the flight time (in seconds). All recordings were made with the same phone and by the same evaluator. The evaluator consistently recorded from the same position (approximately 1 m in height) and at the same distance from the participant (approximately 1.5 m), ensuring a clear view of the participant’s lower limbs. The sagittal plane was used for recording, as it provided a clearer view for identifying the exact take-off and landing frames, compared to a frontal plane view [47]. ( ICC = 0.97) [48].

### Statistical analyses

SPSS Version 26 was used for the statistical analyses. Shapiro-Wilk tests were used to determine the normality of the score distribution (*p* > 0.05). For all the dependent variables, a repeated measures ANOVA was conducted using time (pre-test vs. post-test) and conditions (NHE, DHS, CO) as factors. When condition-time interactions were found, post-hoc t-tests with Bonferroni correction were used to determine the pairwise differences between pre-test to post-test as well as between the post values from the various groups. The effect sizes for all parameters were assessed using partial η^2^. In this context, small, medium, and large effect sizes were represented by partial η^2^ values of 0.02, 0.13, and 0.26, respectively. In addition, to determine the practical significance of the changes between the pre-test and post-test measurements, Cohen’s *d* was calculated. For the interpretation, effect sizes of 0.2, 0.5, and 0.8 or higher in Cohen’s *d* indicated small, medium, and large effect sizes, respectively. The statistical significance of the differences between measurements was evaluated with a significance level of *p* < 0.05.

## Results

**Fig. 2:**
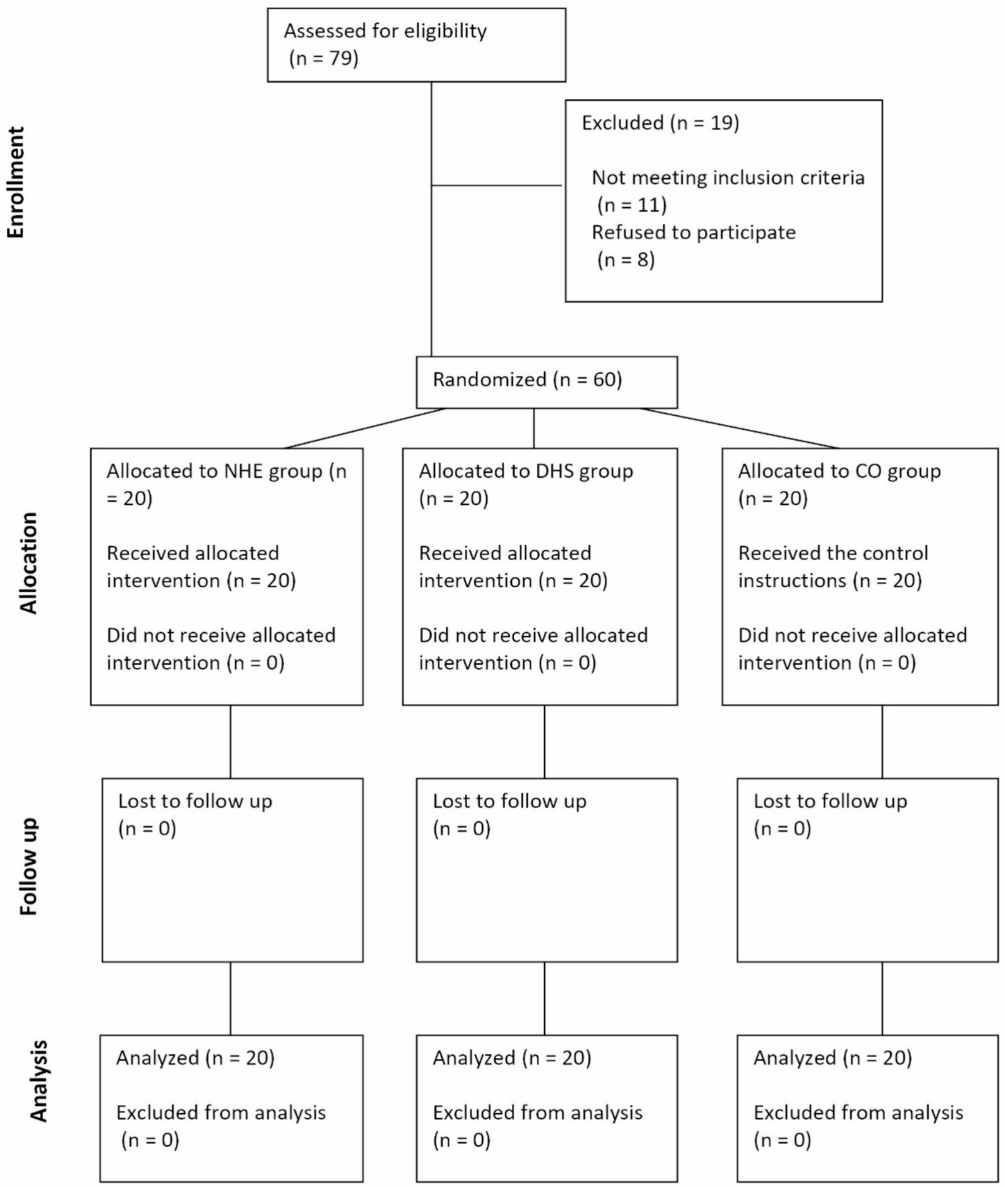
Flowchart of participants throughout the study

**Fig. 3:**
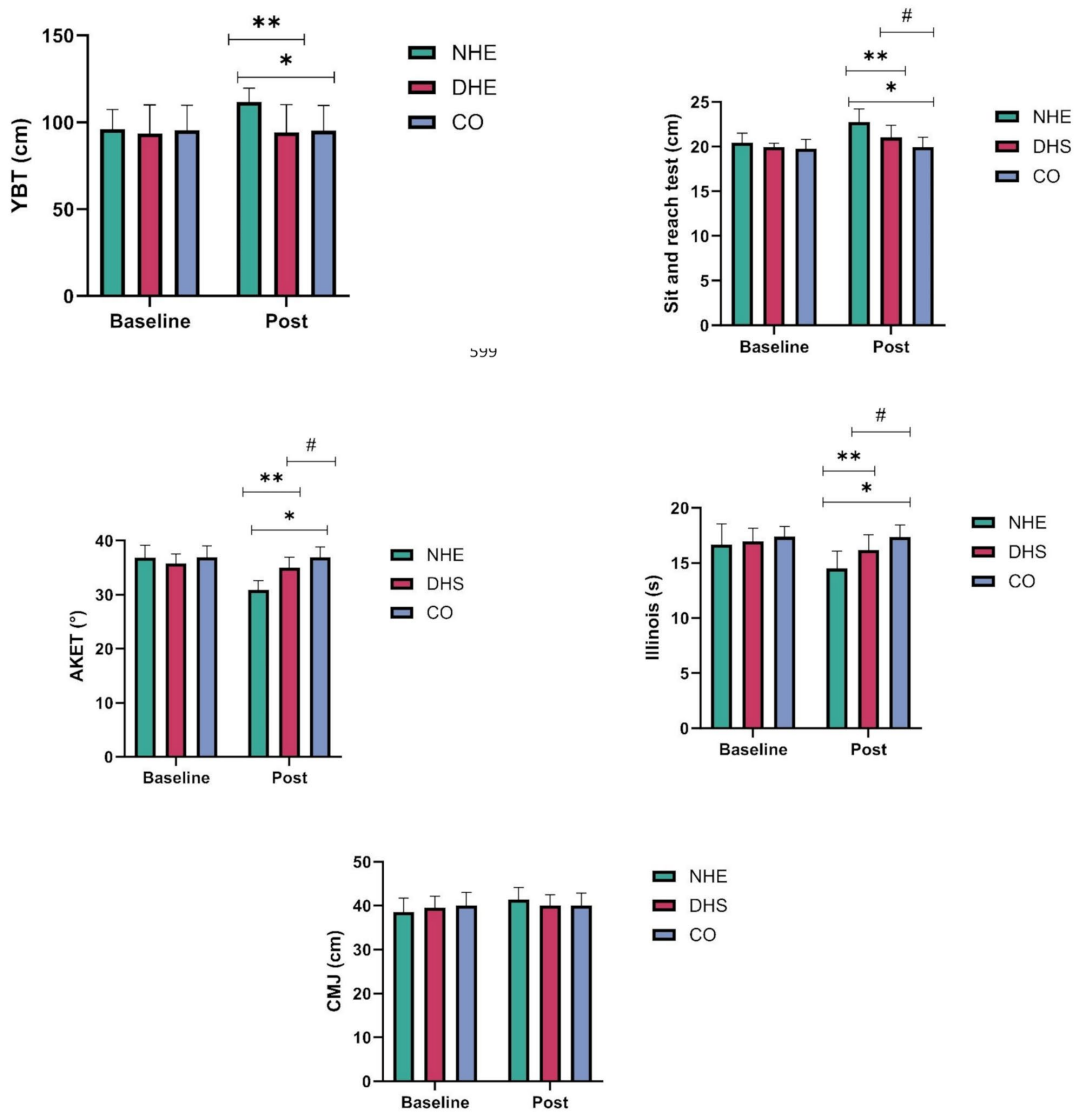
Changes in outcomes between interventions (NHE and DHS) and CO from baseline to post-intervention. NHE = Nordic hamstring exercise, DHS = dynamic hamstring stretching, CO = control group, YBT = Y-balance test, AKET = active knee extension test, CMJ test = countermovement jump test. *= significant difference (*p* < 0.05) between NHE and CO groups, **= significant difference (*p* < 0.05) between NHE and DHS groups, #= significant difference (*p* < 0.05) between DHS and CO groups

Out of the 79 soccer players assessed in the primary stage, 60 met the eligibility criteria (Fig. 2), resulting in an eligibility rate of 75.94%. Remarkably, all 60 eligible participants willingly agreed to participate in the study, reflecting a recruitment rate of 100%. The baseline characteristics of the participants are outlined in Table 2. Among the studied groups, there were no significant differences in the anthropometric characteristics of the participants at baseline assessment. Baseline and post-intervention primary outcome data are presented in Table 3; Fig. 3. The mixed model repeated measures ANOVA demonstrated a significant time effect for the YBT (F(2, 57) = 140.14, *p* = < 0.001), sit-and-reach test (F(2, 57) = 58.03, *p* = < 0.001), AKET (F(2, 57) = 310.71, *p* = < 0.001), Illinois agility run test (F(2, 57) = 57.67, *p* = < 0.001), and CMJ test (F(2, 57) = 79.24, *p* = < 0.001). Moreover, significant interaction effects of group × time were observed for the YBT (F(2, 57) = 129.53, *p* ≤ 0.001, partial η2 = 0.82), sit-and-reach test (F(2, 57) = 15.55, *p* ≤ 0.001, partial η2 = 0.35), AKET (F(2, 57) = 219.18, *p* ≤ 0.001, partial η2 = 0.88), Illinois agility run test (F(2, 57) = 23.46, *p* ≤ 0.001, partial η2 = 0.45), and CMJ test (F(2, 57) = 47.58, *p* ≤ 0.001, partial η2 = 0.62), indicating differential changes over time between the intervention and CO groups for these outcomes. Table 3 shows the post-hoc pairwise comparisons within the groups, indicating significant changes in all parameters in the NHE and DHS groups, but not in the CO group between pre-test to post-test.

**Table 2:** Baseline characteristics of the participants

	NHE	DHS	CO	*p*-value
Age (year)	20.75 (2.48)	21.30 (2.25)	22.35 (2.47)	0.11
Body height (cm)	180.63 (4.31)	177.58 (5.79)	180.13 (8.72)	0.29
Body mass (kg)	72.31 (5.97)	74.10 (3.56)	75.55 (2.41)	0.06
Body mass index (kg/m^2^)	22.25 (2.66)	23.58 (2.15)	23.45 (2.54)	0.17
Experience (year)	7.55 (2.96)	8.35 (3.08)	9.45 (3.51)	0.17
Leg dominance (N)	18 R/2 L	14 R/6 L	17 R/3 L	
Defender (N)	7	8	5	
Midfielder (N)	9	7	9	
Attacker (N)	4	5	6	

Note: NHE = Nordic hamstring exercise, DHS = dynamic hamstring stretching, CO = control. Significance level set as *p* < 0.05

**Table 3:** Comparisons of the outcomes among the studied groups at baseline and post-intervention

Variable	Nordic hamstring exercise					Dynamic hamstring stretching							Control		
	Baseline mean M (SD)	Post mean M (SD)	% change	*p*-value^*^	95% CI	Baseline mean M (SD)	Post mean M (SD)	% change	*p-*value^*^	95% CI	Baseline mean M (SD)	Post mean M (SD)	% change	*p-*value^*^	95% CI
YBT (cm)	96.17 (11.25)	111.80 (7.90)	16.25	< 0.001	-18.41 to -12.85	93.70 (16.34)	94.27 (15.92)	0.60	NS	-1.01 to -0.12	95.40 (14.39)	95.25 (14.45)	−0.15	NS	-0.20 to 0.50
	Cohen’s *d* = 1.61					Cohen’s *d* = 0.03					Cohen’s *d* = 0.01				
Sit-and-reach test (cm)	20.46 (1.05)	22.78 (1.44)	11.33	< 0.001	-3.07 to -1.56	19.94 (0.45)	21.04 (1.35)	5.51	0.005	-1.65 to -0.53	19.79 (1.01)	19.97 (1.11)	0.90	NS	−0.47 to 0.11
	Cohen’s *d* = 1.85					Cohen’s *d* = 1.10					Cohen’s *d* = 0.17				
AKET (°)	36.88 (2.29)	30.92 (1.73)	-16.16	< 0.001	5.28 to 6.63	35.79 (1.79)	35.01 (1.89)	-2.17	< 0.001	0.55 to 1.00	36.90 (2.11)	36.93 (1.90)	0.08	NS	−0.39 to 0.32
	Cohen’s *d* = 2.93					Cohen’s *d* = 0.42					Cohen’s *d* = 0.02				
Illinois agility run test (s)	16.66 (1.89)	14.52 (1.56)	− 12.84	< 0.001	1.50 to 2.76	16.96 (1.21)	16.19 (1.37)	−4.54	0.015	0.30 to 1.23	17.39 (0.93)	17.38 (1.07)	0.05	NS	-0.16 to 0.18
	Cohen’s *d* = 1.23					Cohen’s *d* = 0.59					Cohen’s *d* = 0.01				
CMJ test (cm)	38.57 (3.15)	41.38 (2.77)	7.28	< 0.001	-3.51 to -2.10	39.54 (2.64)	40.04 (2.42)	1.26	< 0.001	-0.75 to -0.24	40.06 (3.01)	40.08 (2.76)	0.04	NS	-0.25 to 0.21
	Cohen’s *d* = 0.94					Cohen’s *d* = 0.19					Cohen’s *d* = 0.006				

Note: M = mean, SD = standard deviation, YBT = Y-balance test, AKET = active knee extension test, CMJ test = countermovement jump test, NHE = Nordic hamstring exercise, DHS = dynamic hamstring stretching, CO = control, NS = not significant, CI = confidence interval

* = indicates significance level based on Bonferroni-adjusted *p*-value results

Furthermore, group comparisons of the post values (Fig. 3) showed significant differences between the NHE and CO groups (*p* = < 0.001, 95% CI: 9.09–24.00) and the NHE and DHS groups (*p* = < 0.001, 95% CI: 9.48–25.58) in the YBT. Moreover, in the sit-and-reach test, significant differences were found between the NHE and CO groups (*p* = < 0.001, 95% CI: 1.00–2.47), the NHE and DHS groups (*p* = 0.001, 95% CI: 0.39–1.86), and the DHS and CO groups (*p* = 0.010, 95% CI: 0.27–1.86). In addition, in the AKET, a significant difference was found between the NHE and CO groups (*p* = < 0.001, 95% CI: -4.49 to − 1.52), the NHE and DHS groups (*p* = 0.047, 95% CI: -2.98 to − 0.01), and the DHS and CO groups (*p* = 0.044, 95% CI: -2.99 to − 0.02). Furthermore, a significant difference was noted in the Illinois agility run test between the NHE and CO groups (*p* = < 0.001, 95% CI: − 2.80 to − 0.79), the NHE and DHS groups (*p* = 0.001, 95% CI: − 2.61 to − 0.72), and the DHS and CO groups (*p* = 0.004, 95% CI: − 1.97 to − 0.39). However, in the CMJ test, no significant differences were observed between the NHE and CO groups (*p* = 0.16, 95% CI: − 0.54 to 3.14), the NHE and DHS groups (*p* = 0.11, 95% CI: − 0.32 to 3.01), and the DHS and CO groups (*p* = 0.95, 95% CI: − 1.78 to 1.69).

## Discussion

The purpose of this study was to determine the effects of an 8-week exercise regimen with either NHE or DHS on balance, ROM, agility, and m in male soccer players with hamstring shortness. The study demonstrated significant improvements in performance, ROM, balance, and agility from the baseline to post-intervention in both interventions (NHE and DHS), but not in the CO group. In addition, it was noted that the participants in the NHE group improved more than those in the DHS and CO groups in terms of balance, ROM, and agility measurements.

The YBT revealed improvements in NHE and DHS, although the NHE group had significantly higher improvements from baseline assessment to post-intervention (16.25%), compared to the DHS (0.60%) and CO (− 0.15%) groups. Unilateral dynamic balance, as assessed with the YBT, is particularly vital for soccer players, as their frequent and powerful movements predominantly involve single-legged actions, including tasks such as accelerating and decelerating, quick cutting maneuvers, kicking, jumping, and landing [49, 50]. Consequently, inadequate unilateral dynamic balance can lead to unsafe conditions for athletes and adversely affect their performance precision [49, 51]. For example, Porrati-Paladino and Cuesta-Barriesu (2021) found that 6 weeks of eccentric exercises improved lower limb stability in female soccer players [52]. Muscle strength and architectural characteristics are adaptable and can be modified by various stimuli, including eccentric strength training [53]. Eccentric training of the hamstrings has been shown to produce neuromuscular adaptations, such as an increase in the length of the fascicle of the long head of the biceps femoris muscle, enhanced muscle strength or volume, and a greater ability to generate higher torque levels at longer muscle lengths [52, 54]. Dynamic stretching is another effective method to address hamstring shortness and enhance unilateral dynamic balance [55]. Dynamic stretching involves active movements that take muscles and joints through their full ROM, thereby increasing muscle temperature and elasticity while also enhancing neuromuscular coordination [56]. This type of stretching is particularly beneficial for athletes, as it prepares the muscles for the specific movements they will perform during training or competition [32]. However, according to our results, it is recommended to use NHE instead of DHS if the goal is to improve YBT performance, and hence likely soccer performance.

Both groups (NHE and DHS) showed an increase in ROM (sit-and-reach test and AKET) following the 8-week training regimen, but again NHE was favorable, compared to DHS. Studies have shown that dynamic stretching can improve muscle flexibility, particularly in the hamstrings, by promoting better muscle activation patterns and increasing joint mobility. In this context, Hegishte et al. (2023) found that PNF and dynamic stretching exercises (three times per week for 4 weeks) resulted in significant improvements in flexibility, balance, and agility in collegiate-level badminton players with hamstring shortness. However, no statistically significant difference between the improvements of dynamic stretching and PNF stretching were found [55]. Paul and Balakrishnan (2015) found that their dynamic stretching protocol (five times per week for 4 weeks) was effective in improving hamstring flexibility in adult males, with an increase from 29.89 degrees to 23.30 degrees in the right leg and from 32.19 degrees to 26.30 degrees in the left leg [57]. Concerning NHE, Medeiros et al. (2020) found improved eccentric strength in the knee flexors, as measured by isokinetic tests (0.68; 95% CI: 0.29–1.06) and NHE tests (1.11; 95% CI: 0.62–1.61). NHE was also beneficial in increasing fascicle length (0.97; 95% CI: 0.46–1.48) [58]. Although a previous study found that decreased strength is one of the weakest risk factors for hamstring injury [59], a prospective cohort study found that elite soccer players with eccentric weakness in the knee flexors (less than 337 N) and a short biceps femoris long head fascicle length (less than 10.56 cm) had a 4.4 and 4.1 times higher risk of sustaining a hamstring strain injury, respectively [60]. Moreover, it has been shown that a 5-week NHE protocol was effective in improving hamstring flexibility, as measured by the sit-and-reach test, from 15.13 ± 4.39 cm to 24.59 ± 3.37 cm among young adults [61]. Therefore, implementing strategies that enhance both eccentric strength and fascicle length is essential to reduce the risk of hamstring strain injuries [58]. Increased fascicle length alters the force-velocity and force-length correlations, having a direct impact on muscular function. In theory, a muscle with longer fascicles contains more in-series aligned sarcomeres, which increases muscle contraction velocity while simultaneously protecting the muscle from injury caused by overlengthening. On the other hand, a muscle with a shorter fascicle length is more at risk for eccentrically produced microscopic muscle injury, which could lead to macroscopic damage. Thus, workouts that increase fascicle length could help to avoid hamstring injury [58, 60].

Considering the Illinois agility run test, our results showed that the NHE group made more significant improvements from baseline assessment to post-intervention (− 12.84%), compared to the DHS (− 4.54%) and CO (0.05%) groups. These results align with the findings of Siddle et al. (2024), who assessed an 8-week low-volume Nordic hamstring curl program’s effect on change of direction ability in elite youth soccer players and observed an improvement in the 5-0-5 test (− 0.06, 95% CI: 0.03–0.09) [62]. Moreover, Chaabene et al. (2019) reported that performing an 8-week NHE intervention effectively improved t-test records (− 1, 90% CI: 0.5–2.2) in young female handball players [63]. Regarding the functional tests (e.g., CMJ test), although no statistically significant differences were observed among the groups post-intervention, practical improvements were noted. The NHE group demonstrated a 7.28% improvement in CMJ performance, accompanied by a large effect size (Cohen’s *d* = 0.94), whereas the DHS group showed a more modest gain of 1.26% with a small effect size (Cohen’s *d* = 0.19). This suggests that the NHE intervention may offer meaningful practical advantages despite the lack of statistical significance. These results are similar to those of Chaabene et al. (2019), who found that CMJ records improved from 23.69 ± 4.66 cm to 27.91 ± 5.25 cm in female handball players who performed an NHE intervention [63]. Hough et al. (2009) also assessed the effects of a dynamic stretching protocol on vertical jump performance in healthy men. They observed a positive impact, with an increase in vertical jump performance after dynamic stretching [64]. Despite the lack of statistical significance in the improvement of CMJ performance in the NHE and DHS groups, the greater than 7% improvement in the NHE group might have practical implications for sports performance. DHS immediately improves performance by activating neuronal and muscular systems [23], whereas NHE offers both immediate and long-term improvements in muscle strength and structure, resulting in greater improvements in explosive actions such as the CMJ [21]. Eccentric training, such as NHEs, develops prolonged neuromuscular adaptations, including improved hamstring strength, consequently improving force generation and injury susceptibility in high-intensity activities [25, 30].

This is the first study that has compared the effects of NHE protocols and dynamic stretching on athletes’ balance, ROM, agility, and performance. We found that NHE is a more efficient approach than DHS to improve the investigated parameters chronically. Indeed, there are distinct physiological mechanisms underlying DHS and NHE that could influence the outcomes. DHS primarily enhances flexibility and ROM through active movement, which can improve muscle elasticity and reduce stiffness [65]. In contrast, NHE targets eccentric strengthening of the hamstrings, which can enhance muscle-tendon unit resilience and improve muscle strength and functional performance [66]. Understanding these mechanisms is crucial for tailoring interventions to specific needs and optimizing training protocols. Future research should also explore these mechanistic differences to better understand how each intervention impacts performance metrics in athletes with hamstring shortness.

This study has some limitations. The parallel-group design may have allowed individual differences in baseline flexibility, motor control, or injury history to influence the results. Although a crossover design could control for such variability, it was not feasible due to the lasting effects of the interventions. Future research should consider strategies to account for these individual differences. This study did not directly evaluate hamstring fascicle length or muscle-tendon unit stiffness, which are critical mechanistic parameters. Moreover, baseline hamstring shortness may have affected the efficacy of dynamic stretching procedures, since limited muscular extensibility might reduce the immediate advantages usually linked to flexibility-oriented approaches. Subsequent research should include direct assessments of structural adaptations, such as ultrasound imaging, and take into consideration a classification of participants based on baseline flexibility to more precisely analyze individual training responses and the mechanisms behind them. Also this study did not account for variables such as sex, sport type, degree of hamstring shortening, or impairments in hamstring length, which future research should consider. Additionally, potential confounding factors, including participants’ injury history and baseline fitness levels, were not controlled and may have influenced the results. Methodologically, while commonly used, the measurement tools (e.g., YBT, sit-and-reach, AKET) may have introduced variability due to differences in administration or their limited ability to capture dynamic flexibility. A more comprehensive assessment, such as 3D motion capture, could provide deeper insights. Lastly, the study lacked a longitudinal follow-up to determine the sustainability of the observed improvements. Future research should incorporate follow-up assessments and consider additional factors like diet, sleep, and stress, which may impact training outcomes.

## Conclusions

This study demonstrated that both NHE and DHS can improve performance, ROM, balance, and agility in soccer players with hamstring shortness. However, NHE was found to be a more effective intervention for enhancing these parameters. Specifically, NHE produced more significant improvements in balance, range of motion, and agility compared to DHS. Based on these findings, it is recommended that NHE be implemented as a key component in training regimens for soccer players with hamstring shortness, particularly to target dynamic balance and hamstring flexibility. Coaches and trainers could consider incorporating NHE exercises, such as Nordic hamstring curls, into weekly training sessions to increase eccentric strength, fascicle length, and overall hamstring resilience. This approach may help reduce the risk of hamstring injuries while also improving functional performance on the field, such as acceleration, deceleration, and change of direction. Additionally, future research should explore optimal dosing and duration of NHE to maximize its benefits for specific player needs.

## Electronic supplementary material

Below is the link to the electronic supplementary material.

Supplementary Material 1

## Data Availability

The data that supports the findings of this study are available in the Zenodo data repository at 10.5281/zenodo.15265634.

## Abbreviations

AKET: Active Knee Extension Test
CMJ: Countermovement Jump Test
CO: Control Group
DHS: Dynamic Hamstring Stretching
IAT: Illinois Agility Run Test
NHE: Nordic Hamstring Exercise
PNF: Proprioceptive Neuromuscular Facilitation
ROM: Range of Motion
SR: Sit-and-Reach Test
YBT: Y-Balance Test

## References

[1] Agur AM, Dalley AF. Moore’s essential clinical anatomy. Lippincott Williams & Wilkins; 2018.

[2] Chumanov ES, Heiderscheit BC, Thelen DG. The effect of speed and influence of individual muscles on hamstring mechanics during the swing phase of sprinting. J Biomech. 2007;40(16):3555–62. 17659291 10.1016/j.jbiomech.2007.05.026

[3] Espejo-Antúnez L, Carracedo-Rodríguez M, Ribeiro F, Venâncio J, De la Cruz-Torres B, Albornoz-Cabello M. Immediate effects and one-week follow-up after neuromuscular electric stimulation alone or combined with stretching on hamstrings extensibility in healthy football players with hamstring shortening. J Bodyw Mov Ther. 2019;23(1):16–22. 30691745 10.1016/j.jbmt.2018.01.017

[4] Fereydounnia S, Shadmehr A, Salemi P. Acute effect of inhibitory kinesio tape on range of motion, dynamic balance, and gait in athletes with hamstring shortness. Foot. 2022;53:101925. 36037779 10.1016/j.foot.2022.101925

[5] Kim D-H, Lee JJ. Effects of instrument-assisted soft tissue mobilization technique on strength, knee joint passive stiffness, and pain threshold in hamstring shortness. J Back Musculoskelet Rehabil. 2018;31(6):1169–76. 30040707 10.3233/BMR-170854

[6] García-Pinillos F, Ruiz-Ariza A, Moreno del Castillo R, Latorre-Román PÁ. Impact of limited hamstring flexibility on vertical jump, kicking speed, sprint, and agility in young football players. J Sports Sci. 2015;33(12):1293–7. 25761523 10.1080/02640414.2015.1022577

[7] Maras G, Arikan H, Citaker S. Comparison of the effects of 4-week instrument assisted soft tissue mobilization and static stretching on strength, ROM, flexibility, and painthreshold in hamstring muscle shortness. J Bodyw Mov Ther. 2024;40(1):575–58 10.1016/j.jbmt.2024.05.008 39593646

[8] Kellis E, Blazevich AJ. Hamstrings force-length relationships and their implications for angle-specific joint torques: a narrative review. BMC Sports Sci Med Rehabilitation. 2022;14(1):166. 10.1186/s13102-022-00555-6 PMC9446565 36064431

[9] Trainor TJ, Trainor MA. Etiology of low back pain in athletes. Curr Sports Med Rep. 2004;3:41–6. 14728913 10.1249/00149619-200402000-00008

[10] Watsford ML, Murphy AJ, McLachlan KA, Bryant AL, Cameron ML, Crossley KM, et al. A prospective study of the relationship between lower body stiffness and hamstring injury in professional Australian rules footballers. Am J Sports Med. 2010;38(10):2058–64. 20595555 10.1177/0363546510370197

[11] Ali A. Measuring soccer skill performance: a review. Scand J Med Sci Sports. 2011;21(2):170–83. 21210855 10.1111/j.1600-0838.2010.01256.x

[12] Ardern CL, Pizzari T, Wollin MR, Webster KE. Hamstrings strength imbalance in professional football (Soccer) players in Australia. J Strength Conditioning Res. 2015;29(4). 10.1519/JSC.0000000000000747 25426513

[13] Ekstrand J, Hägglund M, Waldén M. Injury incidence and injury patterns in professional football: the UEFA injury study. Br J Sports Med. 2011;45(7):553–8. 19553225 10.1136/bjsm.2009.060582

[14] Mendiguchia J, Alentorn-Geli E, Brughelli M. Hamstring strain injuries: are we heading in the right direction? Br J Sports Med. 2012;46(2):81–5. 21677318 10.1136/bjsm.2010.081695

[15] Freckleton G, Pizzari T. Risk factors for hamstring muscle strain injury in sport: a systematic review and meta-analysis. Br J Sports Med. 2013;47(6):351–8. 22763118 10.1136/bjsports-2011-090664

[16] Liu H, Garrett WE, Moorman CT, Yu B. Injury rate, mechanism, and risk factors of hamstring strain injuries in sports: a review of the literature. J Sport Health Sci. 2012;1(2):92–101.

[17] Van Beijsterveldt A, van de Port IG, Vereijken A, Backx F. Risk factors for hamstring injuries in male soccer players: a systematic review of prospective studies. Scand J Med Sci Sports. 2013;23(3):253–62. 22724435 10.1111/j.1600-0838.2012.01487.x

[18] Alizadeh S, Daneshjoo A, Zahiri A, Anvar SH, Goudini R, Hicks JP, et al. Resistance training induces improvements in range of motion: a systematic review and meta-analysis. Sports Med. 2023;53(3):707–22. 36622555 10.1007/s40279-022-01804-x PMC9935664

[19] Konrad A, Nakamura M, Tilp M, Donti O, Behm DG. Foam rolling training effects on range of motion: a systematic review and Meta-Analysis. Sports Med. 2022;52(10):2523–35. 35616852 10.1007/s40279-022-01699-8 PMC9474417

[20] Konrad A, Alizadeh S, Daneshjoo A, Anvar SH, Graham A, Zahiri A et al. Chronic effects of stretching on range of motion with consideration of potential moderating variables: a systematic review with meta-analysis. J Sport Health Sci. 2023;13(2):186–94 10.1016/j.jshs.2023.06.002 PMC10980866 37301370

[21] Borges MO, Medeiros DM, Minotto BB, Lima CS. Comparison between static stretching and proprioceptive neuromuscular facilitation on hamstring flexibility: systematic review and meta-analysis. Eur J Physiotherapy. 2018;20(1):12–9.

[22] Medeiros DM, Cini A, Sbruzzi G, Lima CS. Influence of static stretching on hamstring flexibility in healthy young adults: systematic review and meta-analysis. Physiother Theory Pract. 2016;32(6):438–45. 27458757 10.1080/09593985.2016.1204401

[23] Rizvi FR, Rasheed N, Simon NH, Chatterjee A. The effect of static stretching and PNF hold-relax stretching on increasing flexibility of shortened hamstring muscle among sedentary living female students-randomized controlled trial. Int J Sci Res. 2020;9(11):157–61.

[24] Behm DG, Alizadeh S, Daneshjoo A, Konrad A. Potential effects of dynamic stretching on injury incidence of athletes: a narrative review of risk factors. Sports Med. 2023;53(7):1359–73. 37162736 10.1007/s40279-023-01847-8 PMC10289929

[25] Takeuchi K, Nakamura M, Konrad A, Mizuno T. Long-term static stretching can decrease muscle stiffness: a systematic review and meta-analysis. Scand J Med Sci Sports. 2023;33(8):1294–306. 37231582 10.1111/sms.14402

[26] Ribeiro-Alvares JB, Marques VB, Vaz MA, Baroni BM. Four weeks of nordic hamstring exercise reduce muscle injury risk factors in young adults. J Strength Conditioning Res. 2018;32(5):1254–62. 10.1519/JSC.0000000000001975 28459795

[27] Delvaux F, Schwartz C, Decréquy T, Devalckeneer T, Paulus J, Bornheim S, et al. Influence of a field hamstring eccentric training on muscle strength and flexibility. Int J Sports Med. 2020;41(04):233–41. 31935778 10.1055/a-1073-7809

[28] Vogt M, Hoppeler HH. Eccentric exercise: mechanisms and effects when used as training regime or training adjunct. J Appl Physiol. 2014;116(11):1446–54 10.1152/japplphysiol.00146.2013 24505103

[29] Gérard R, Gojon L, Decleve P, Van Cant J. The effects of eccentric training on biceps femoris architecture and strength: a systematic review with meta-analysis. J Athl Train. 2020;55(5):501–14. 32216654 10.4085/1062-6050-194-19 PMC7249279

[30] Goode AP, Reiman MP, Harris L, DeLisa L, Kauffman A, Beltramo D, et al. Eccentric training for prevention of hamstring injuries may depend on intervention compliance: a systematic review and meta-analysis. Br J Sports Med. 2015;49(6):349–56. 25227125 10.1136/bjsports-2014-093466

[31] Van der Horst N, Smits D-W, Petersen J, Goedhart EA, Backx FJ. The preventive effect of the nordic hamstring exercise on hamstring injuries in amateur soccer players: a randomized controlled trial. Am J Sports Med. 2015;43(6):1316–23. 25794868 10.1177/0363546515574057

[32] Alimoradi M, Sahebozamani M, Hosseini E, Konrad A, Noorian S. The effect on flexibility and a variety of performance tests of the addition of 4 weeks of soleus stretching to a regular dynamic stretching routine in amateur female soccer players. Sports. 2023;11(7). 10.3390/sports11070138 PMC10383580 37505625

[33] Yıldırım MŞ, Tuna F, Kabayel DD, Süt N. The cut-off values for the diagnosis of hamstring shortness and related factors. Balkan Med J. 2018;35(5):388–93. 29914231 10.4274/balkanmedj.2017.1517 PMC6158462

[34] Lee JH, Jang K-M, Kim E, Rhim HC, Kim H-D. Effects of static and dynamic stretching with strengthening exercises in patients with patellofemoral pain who have inflexible hamstrings: a randomized controlled trial. Sports Health. 2021;13(1):49–56. 32790575 10.1177/1941738120932911 PMC7734366

[35] Chen CH, Xin Y, Lee KW, Lin MJ, Lin JJ. Acute effects of different dynamic exercises on hamstring strain risk factors. PLoS ONE. 2018;13(2):e0191801. 29390001 10.1371/journal.pone.0191801 PMC5794078

[36] Iwata M, Yamamoto A, Matsuo S, Hatano G, Miyazaki M, Fukaya T, et al. Dynamic stretching has sustained effects on range of motion and passive stiffness of the hamstring muscles. J Sports Sci Med. 2019;18(1):13. 30787647 PMC6370952

[37] Bukry SA, Raja Azidin MRF, Justine M, Manaf H. The effects of short-duration high-intensity soccer fatigue simulation on dynamic balance and lower limb isokinetic strength in youth soccer players. Ann-Appl-Sport-Sci. 2022;10(2):0.

[38] Pardos-Mainer E, Casajús JA, Gonzalo-Skok O. Adolescent female soccer players’ soccer-specific warm-up effects on performance and inter-limb asymmetries. Biology Sport. 2019;36(3):199–207. 10.5114/biolsport.2019.85453 PMC6786331 31624413

[39] Xixirry MG, Riberto M, Manoel LS. Analysis of Y balance test and dorsiflexion lunge test in professional and amateur soccer players. Revista Brasileira de Medicina do Esporte. 2019;25.

[40] Greenberg ET, Barle M, Glassman E, Jacob L, Jaafar H, Johnson A, et al. Reliability and stability of the Y balance test in healthy early adolescent female athletes. Orthop J Sports Med. 2019;7(3suppl):2325967119S00051. PMC6449012 30997273

[41] Jo S-H, Choi H-J, Cho H-S, Yoon J-H, Lee W-Y. Effect of core balance training on muscle tone and balance ability in adult men and women. Int J Environ Res Public Health. 2022;19(19):12190. 36231489 10.3390/ijerph191912190 PMC9564429

[42] Kage V, Ratnam R. Immediate effect of active release technique versus mulligan bent leg raise in subjects with hamstring tightness: a randomized clinical trial. Int J Physiother Res. 2014;2(1):301–4.

[43] Henriques-Neto D, Minderico C, Peralta M, Marques A, Sardinha LB. Test–retest reliability of physical fitness tests among young athletes: the FITescola^®^ battery. Clin Physiol Funct Imaging. 2020;40(3):173–82. 32056351 10.1111/cpf.12624

[44] Niewiadomy P, Szuścik-Niewiadomy K, Kochan M, Kuszewski MT. The relationship between active and passive flexibility of the knee flexors. Muscles Ligaments Tendons J (MLTJ). 2021;11(2).

[45] Hachana Y, Chaabène H, Nabli MA, Attia A, Moualhi J, Farhat N, et al. Test-retest reliability, criterion-related validity, and minimal detectable change of the Illinois agility test in male team sport athletes. J Strength Conditioning Res. 2013;27(10):2752–9. 10.1519/JSC.0b013e3182890ac3 23439329

[46] Huang S, Zhang H-J, Wang X, Lee WC-C, Lam W-K. Acute effects of soleus stretching on ankle flexibility, dynamic balance and speed performances in soccer players. Biology. 2022;11(3):374. 35336748 10.3390/biology11030374 PMC8945810

[47] Bogataj Š, Pajek M, Andrašić S, Trajković N. Concurrent validity and reliability of my jump 2 app for measuring vertical jump height in recreationally active adults. Appl Sci. 2020;10(11). 10.3390/ijerph17103708 PMC7277223 32466091

[48] Rago V, Brito J, Figueiredo P, Carvalho T, Fernandes T, Fonseca P et al. Countermovement jump analysis using different portable devices: implications for field testing. Sports. 2018;6(3). 10.3390/sports6030091 PMC6162675 30200384

[49] López-Valenciano A, Ayala F, Puerta JM, Croix MDS, Vera-García F, Hernández-Sánchez S, et al. A preventive model for muscle injuries: a novel approach based on learning algorithms. Med Sci Sports Exerc. 2018;50(5):915. 29283933 10.1249/MSS.0000000000001535 PMC6582363

[50] Denerel N, Ergün M, Yüksel O, Özgürbüz C, Karamızrak O. The acute effects of static and dynamic stretching exercises on dynamic balance performance. Spor Hekimliği Dergisi. 2019;54(3):148–57.

[51] Ayub F, Naseer A, Javed S. Role of agility and dynamic balance in performance of university football players of pakistan. THE SPARK A HEC recognized. Journal. 2019;4:181–9.

[52] Porrati-Paladino G, Cuesta-Barriuso R. Effectiveness of plyometric and eccentric exercise for jumping and stability in female soccer players—a single-blind, randomized controlled pilot study. Int J Environ Res Public Health. 2021;18(1):294. 33401532 10.3390/ijerph18010294 PMC7796027

[53] Mjølsnes R, Arnason A, Østhagen T, Raastad T, Bahr R. A 10-week randomized trial comparing eccentric vs. concentric hamstring strength training in well‐trained soccer players. Scand J Med Sci Sports. 2004;14(5):311–7. 15387805 10.1046/j.1600-0838.2003.367.x

[54] Opar DA, Williams MD, Timmins RG, Hickey J, Duhig SJ, Shield AJ. Eccentric hamstring strength and hamstring injury risk in Australian footballers. Med Sci Sports Exerc. 2015;47(4):857–65. 25137368 10.1249/MSS.0000000000000465

[55] Hegishte AS, Kumar N. Effect of proprioceptive neuromuscular facilitation and dynamic stretching on flexibility, agility, and balance in hamstring tightness among collegiate level badminton players. Int J Res Med Sci. 2023;11(5):1758–63.

[56] Opplert J, Babault N. Acute effects of dynamic stretching on muscle flexibility and performance: an analysis of the current literature. Sports Med. 2018;48:299–325. 29063454 10.1007/s40279-017-0797-9

[57] Paul J, Balakrishnan P. Comparative effect of static and dynamic stretching exercise to improve flexibility of hamstring muscles among male adults. IJMAES. 2015;1(2):53–8.

[58] Medeiros DM, Marchiori C, Baroni BM. Effect of nordic hamstring exercise training on knee flexors eccentric strength and fascicle length: a systematic review and meta-analysis. J Sport Rehabilitation. 2020;30(3):482–91. 10.1123/jsr.2019-0388 33049705

[59] Van Dyk N, Bahr R, Whiteley R, Tol JL, Kumar BD, Hamilton B, et al. Hamstring and quadriceps isokinetic strength deficits are weak risk factors for hamstring strain injuries: a 4-year cohort study. Am J Sports Med. 2016;44(7):1789–95. 27002102 10.1177/0363546516632526

[60] Timmins RG, Shield AJ, Williams MD, Lorenzen C, Opar DA. Architectural adaptations of muscle to training and injury: a narrative review outlining the contributions by fascicle length, pennation angle and muscle thickness. Br J Sports Med. 2016;50(23):1467–72. 26817705 10.1136/bjsports-2015-094881

[61] Babu SK, Paul A. Effectiveness of nordic hamstring exercise in improving hamstring muscle flexibility, strength and endurance among young adults. System. 2018;3:4.

[62] Siddle J, Weaver K, Greig M, Harper D, Brogden CM. A low-volume nordic hamstring curl programme improves change of direction ability, despite no architectural, strength or speed adaptations in elite youth soccer players. Res Sports Med. 2024;32(1):49–60. 35642790 10.1080/15438627.2022.2079984

[63] Chaabene H, Negra Y, Moran J, Prieske O, Sammoud S, Ramirez-Campillo R, et al. Effects of an eccentric hamstrings training on components of physical performance in young female handball players. Int J Sports Physiol Perform. 2020;15(1):91–7. 31034308 10.1123/ijspp.2019-0005

[64] Hough PA, Ross EZ, Howatson G. Effects of dynamic and static stretching on vertical jump performance and electromyographic activity. J Strength Conditioning Res. 2009;23(2):507–12. 10.1519/JSC.0b013e31818cc65d 19204571

[65] Cai P, Liu L, Li H. Dynamic and static stretching on hamstring flexibility and stiffness: a systematic review and meta-analysis. Heliyon. 2023;9(8):e18795 10.1016/j.heliyon.2023.e18795 PMC10407730 37560703

[66] Liang F, Hongfeng H, Ying Z. The effects of eccentric training on hamstring flexibility and strength in young dance students. Sci Rep. 2024;14(1):3692. 38355663 10.1038/s41598-024-53987-0 PMC10866893

