# Supplementary material for: The effects of 8 weeks of dynamic hamstring stretching or nordic hamstring exercises on balance, range of motion, agility, and muscle performance among male soccer players with hamstring shortness: a randomized controlled trial
Source: BMC Sports Sci Med Rehabil. 2025 Jul 9;17:187. doi: 10.1186/s13102-025-01216-0 (PMC12239349; doi:10.1186/s13102-025-01216-0)
Supplement: Supplementary file 1 — Supplementary Material 1 [file 13102_2025_1216_MOESM1_ESM.docx]

| **Day** | **Session type** | **Duration** | **Content** |
| --- | --- | --- | --- |
| Saturday | Intervention + team training | ~100–105 min | * NHE or DHS (10–15 min) * Warm-up, technical drills, small-sided games |
| Sunday | Intervention + team training | ~100–105 min | * NHE or DHS (10–15 min) * Tactical play, formations, game scenarios, light conditioning |
| Monday | Rest or recovery | - | No structured physical activity |
| Tuesday | Intervention + team training | ~100–105 min | * NHE or DHS (10–15 min) * Technical and tactical drills |
| Wednesday | Intervention + team training | ~100–105 min | * NHE or DHS (10–15 min) * Match simulation, offensive/defensive drills, set pieces |
| Thursday | Intervention + team training | ~100–105 min | * NHE or DHS (10–15 min) * Technical drills, small-sided games |
| Friday | Rest or recovery | - | No structured physical activity |

Supplementary Table 1. Weekly training schedule during the 8-week intervention period.

Note: NHE = Nordic hamstring exercise, DHS = dynamic hamstring stretching.
